# Muscle wasting in the ICU can be reliably monitored using ultrasound

**DOI:** 10.1186/cc12377

**Published:** 2013-03-19

**Authors:** H Jørgensen, B Nedergaard, T Gilsaa

**Affiliations:** 1 Lillebaelt Hospital, Kolding, Denmark; 2 Odense University Hospital, Svendborg, Denmark

## Introduction

The aim of this study was to establish the intraobserver and interobserver variation of ultrasonographic measurements of the rectus femoris muscle cross-section area (RF-CSA). Muscle wasting is frequent in the ICU, affecting more than one-half of the patients with severe sepsis [1]. Muscle mass reduces rapidly, and 15 to 20% is lost within the first week [1]. To monitor muscle mass, ultrasound has the benefits of being both readily available in the ICU and non-invasive. Ultrasonographic measurement of RF-CSA has an almost perfect correlation with MRI (mean interclass correlation (ICC) = 0.999) [2] and RF-CSA is linearly related to maximum voluntary contraction strength in both healthy subjects and COPD patients (*r *= 0.78) [3].

## Methods

The study had two purposes: to determine the intraobserver variation for RF-CSA by one observer scanning 15 healthy adult volunteers three times each at 2-day intervals; and to determine the interobserver variation for RF-CSA by two observers each scanning 15 adult ICU patients on the same day. Patients were in a supine position, legs in passive extension. The transducer was placed perpendicular to the long axis of the right thigh over the RF, two-thirds of the distance from the anterior superior iliac spine to the superior patellar border [1]. RF-CSA was calculated by planimetry. At each scan, three measurements were made. For intraobserver variation, the 3 × 3 scans were analyzed using the interclass correlation coefficient. For interobserver variation, the three measurements from each observer were averaged and compared using Bland-Altman statistics.

## Results

Intraobserver variation: 15 healthy adults, age 39.6 ± 2.4 years, weight 66.8 ± 2.3 kg, sex three male/12 female. ICC: 0.996 (95% CI: 0.990 to 0.998). Interobserver variation: 15 ICU patients, age: 77 ± 8.3 years, weight: 71.3 ± 9.1 kg, sex nine male/six female. Bland-Altman: bias: -0.07 cm^2^, 95% limits of agreement -0.188 to 0.048 cm^2^.

## Conclusion

Ultrasonographic measurement of RF-CSA is easily learned and quickly performed. It has a very low intraobserver and interobserver variation and can be recommended as a reliable method for monitoring muscle wasting in the ICU.
